# Pregnancy complicated by HTLV‐1‐associated myelopathy/tropical spastic paraparesis (HAM/TSP): a case report

**DOI:** 10.1002/ccr3.4511

**Published:** 2021-07-23

**Authors:** Hiroshi Mori, Eiji Shibata, Tomoichiro Kuwazuru, Takayuki Uchimura, Emi Kondo, Yukio Iwanaka, Kiyoshi Yoshino

**Affiliations:** ^1^ Department of Perinatal Medical Center School of Medicine University of Occupational and Environmental Health Kitakyushu Japan; ^2^ Department of Microbiology Graduate School of Medicine University of Occupational and Environmental Health Kitakyushu Japan; ^3^ Department of Obstetrics and Gynecology School of Medicine University of Occupational and Environmental Health Kitakyushu Japan; ^4^ Department of Neurology School of Medicine University of Occupational and Environmental Health Kitakyushu Japan

**Keywords:** HTLV‐1, HTLV‐1‐associated myelopathy, pregnancy, tropical spastic paraparesis

## Abstract

Pregnancy was not associated with deterioration of HAM nor was HAM associated with adverse pregnancy outcome in this case. These findings suggest that women with HAM/TSP, even those who use a wheelchair, should not be discouraged from pregnancy.

## Graphical abstract

Pregnancy was not associated with deterioration in HAM nor was HAM associated with adverse pregnancy outcome in this case. These findings suggest that women with HAM/TSP, even those who use a wheelchair, should not be discouraged from pregnancy.

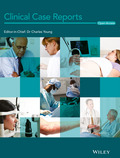

## 1 INTRODUCTION

There are few reports on pregnancy and childbirth of HTLV‐1‐associated myelopathy/tropical spastic paraparesis (HAM/TSP) patients. We present a woman who diagnosed with HAM/TSP and used a wheelchair in daily life. She had no neurological impairments and no obstetrical complications during her three courses of pregnancy and childbirth.

Five to ten million people were infected with the human T‐cell leukemia virus type 1 (HTLV‐1) throughout the world.^1^ HTLV‐1‐associated myelopathy/tropical spastic paraparesis (HAM/TSP) is developed by approximately 0.25% to 3.8% of HTLV‐1 carriers.^2^ HAM/TSP is a slowly progressive type of myelopathy and is characterized by spastic paraparesis, bladder and sphincter dysfunction, and mild sensory impairment. Approximately 80% of HAM progresses in patients slowly after the onset. A rapid progression of HAM/TSP in nearly 20% of patients causes walking abnormalities that occurred within 2 years and they use wheelchair in daily life. The average age of onset is recorded around 50 years.^3^ A nationwide HTLV‐1 screening program for pregnant women started in 2011, and provision of information to HTLV‐1 careers has been systematized in Japan.^4^ However, there is little information on pregnancy and childbirth of patients with HAM/TSP developed from HTLV‐1 carriers.^5^

There are more female patients with HAM than male patients, and several medical articles mentioned about early juvenile HAM/TSP.^6^ The purpose of this study was to provide a report of pregnancy and childbirth of patients with HAM/TSP and to develop an appropriate perinatal care for women with HAM/TSP in reproductive age, their families, and medical staffs. We report here of one woman with HAM/TSP and her three‐time consecutive courses of pregnancy and childbirth without severe complications on her and her baby. These findings imply female patients with HAM/TSP including patients who are wheelchair‐dependent in daily life should not be discouraged from pregnancy.

## 2 CASE HISTORY

### 2.1 History of present illness

Written informed consent was obtained to report the case. This patient felt inguinal region abnormality while exercising when she was 15‐year‐old. Her lower limbs were gradually changed harder to move. She had difficulty in walking and presented to the neurology department at the age of 19. The patient had a family history of HTLV‐1‐associated diseases, and her maternal grandmother was diagnosed with adult T‐cell leukemia (ATL). Upon sensory examination, the lower limbs were shown normal and there was an absence of neurological abnormalities in the upper limb.

She was tested positive for serum anti‐HTLV‐1 antibody. Cerebrospinal fluid test showed anti‐HTLV‐1 antibody (detected at 1:512 dilution) and increased IgG index.

The patient had an MRI scan of the cervical and thoracic spine to rule out other spinal diseases (eg, hereditary spastic paraplegia, myelitis, and multiple sclerosis).

Based on the physical examination, laboratory tests, imaging tests, she was diagnosed with HAM/TSP.

At the age of 20, one year after the initial diagnosis, an aggravation of gait disorder appeared and steroid pulse therapy was performed with a diagnosis of acute exacerbation. The patient maintained a stable gait condition and was followed up as an outpatient.

Her symptoms of spastic paraplegia were gradually worsened. She was unable to maintain a gait stability and required a wheelchair in daily life after 2 years of her initial diagnosis. The patient had dysuria symptoms at the same time, and self‐intermittent catheterization for bladder dysfunction was performed. She had admitted for a urinary tract infection (UTI) twice before her first pregnancy.

### 2.2 Courses of pregnancy and childbirth

A 24‐year‐old, G1P0, pregnant woman diagnosed with HAM/TSP presented at our hospital at 12 weeks of gestation. On her neurological physical findings of bilateral lower limbs at the first visit, she was diagnosed spastic paraparesis, limb tendon reflex hyperreflexia, and Babinski's reflex positive. No neurological abnormalities were found in the upper limbs. Neurological disability was assessed using Osame's Motor Disability Score (OMDS)^7^ and recorded 11 scores: unable to move and turn over on her own on the bed. Her prescription medications included as follows: prednisolone; baclofen; eperisone; hydrochloride; mecobalamin; folic acid; alfacalcidol; and magnesium oxide. We decided to continue these medications during pregnancy. There were no obstetric abnormalities and no exacerbation of neurological findings in the first course of pregnancy.

The patient was moderately using compression pantyhose in daily life. Her husband who is a physical therapist conducted her therapy for the prevention of venous thromboembolism (VTE) during pregnancy. The patient did not stay the condition of immobility; however, she was using a wheelchair in daily life. Regarding the fact that there is no treatment for VTE before first course of pregnancy and no other risk factors, we considered that the patient did not require anticoagulation agents such as prophylactic dose of low molecular weight heparin during pregnancy.

Constipation trouble caused during pregnant period. A UTI was not admitted during her course of pregnancy and childbirth.

At 37 weeks and 5 days of gestation, the patient was admitted to our hospital because of spontaneous labor with cervical dilation of 5 cm. We performed augmentation of labor with the use of intravenous oxytocin for the treatment of prolonged labor in the first stage of labor. The prolonged labor continued in the second stage of labor. The patient was evaluated for failure to proceed with childbirth by herself, and obstetricians performed vacuum extraction delivery. The neonate was 3,128 g with umbilical cord pH of 7.25, and the 1‐minute and 5‐minute Apgar scores were 8/9. Her labor lasts for 21 h, and postpartum blood loss was estimated at 686 ml.

In postpartum period, the patient had a treatment as per current standard non‐pharmacological postpartum VTE prophylaxis recommendations; furthermore, she was using a compression pantyhose and was conducted physical therapy by her husband. VTE did not occur during the pregnancy, peripartum, and postpartum periods.

The baby was breast‐fed, and fed with formula milk for three months to prevent the transmission of HTLV‐1 via breast‐feeding.

Two times of consecutive pregnancies after the first pregnancy and childbirth were stable. The summary of these three consecutive pregnancies is presented in Table 1.

**TABLE 1:** Delivery outcomes and postpartum management

	The first pregnancy and childbirth	The second pregnancy and childbirth	The third pregnancy and childbirth
Maternal age (year)	24	26	27
Type of conception (natural or ART)	Natural	Natural	Natural
Gestational age	38w6d	37w4d	37w6d
Neonatal birth weight (g)	3128(+0.1SD)	2874(+0.4SD)	2732(−0.5SD)
Neonatal birth height (cm)	50.5(+1.0SD)	49(+0.6SD)	48(±0.0SD)
Apgar score (1/5 min)	8/9	9/9	8/9
Umbilical artery (pH)	7.250	7.374	7.331
Labor time (h)	21	5.5	5.5
Mode of delivery	VE	NVD	NVD
Postpartum blood loss (ml)	686	202	168
Nutrition	FM after STBF	FM after STBF	FM only
VTE prevention	GCS and PT	GCS and PT	GCS and PT

Abbreviations: ART, assisted reproductive technologies; FM, formula milk; GCS, graduated compression stocking; NVD, normal vaginal delivery; PT, physical therapy; STBF, short‐term (3 months) breast‐feeding; VE, vacuum extraction; VTE, venous thromboembolism.

### 2.3 Outcome and follow‐up

There was no impairment of neurological findings after three times of pregnancy and childbirth. The patient continued breast‐feeding and formula milk for her second baby by short‐term considering the prevention of a transmission of HTLV‐1 via breast‐feeding. Her third baby was fed by formula milk only. The babies were monitored for the infection until three‐year‐old, and they have not been infected with HTLV‐1 so far.

## 3 DISCUSSION

There is no evidence‐based medical literature on managing pregnancy and childbirth for pregnancies complicated by HAM/TSP. Regarding pregnancy and childbirth management of patients with HAM/TSP, we have to consider the following: (1) fertility, (2) the impact of pregnancy and childbirth on the disease, (3) the impacts of the disease on the course of pregnancy and childbirth, (4) prevention of mother‐to‐child HTLV‐1 transmission, and (5) maternal VTE prophylaxis.

### 3.1 Fertility

There are limited data about the impact of HTLV‐1 on fertility. In some reports, no differences were found in fertilization among the prevalence of HTLV‐1 infection.^8^ Additionally, no data of the impact on fertility of patients with HAM/TSP were found.

### 3.2 The impact of pregnancy and childbirth on the disease

Although the studies on the effect of HAM/TSP on pregnant women are scarce, it is found that there is a potential impact of HAM/TSP on pregnancy. High HTLV‐1 proviral load (PVL) levels have been evaluated as important predictors of development of ATL,^9^ and HTLV‐1 PVL levels reach plateau during pregnancy and increase after delivery.^10^ One case of a postpartum deterioration of HAM/TSP was reported from Japan.^11^ In this case, the patient's HTLV‐1 PVL levels were unrecorded. It is unclear that whether postpartum HTLV‐1 PVL levels elevation is transient or persistent at this moment. This patient had three consecutive pregnancies and childbirth within four years; however, the risk of subsequent pregnancy shortly after giving birth is not revealed.

### 3.3 The impact of the disease on the course of pregnancy and childbirth

Constipation, dysuria, and gait disturbance possibly have a negative effect on pregnancy and childbirth. These symptoms can be occurred by the physical and physiological changes: uterine enlargement, maternal weight gain, low levels of intestinal peristalsis due to endocrinological changes. Neurogenic bladder is commonly observed in HAM/TSP, which results in increased UTI rates. Therefore, in order to prevent preterm birth, it is necessary to be diagnosed early with a physical examination and microscopic examination of urine.

### 3.4 Prevention of mother‐to‐child HTLV‐1 transmission

The long period of breast‐feeding is connected to the risk of HTLV‐1 infection. For the purpose of preventing transmission from the mother to child, a patient and her family are expected to know about the risk. In case the patient requested breast‐feeding, based on some of the retrospective studies, the HTLV‐1 carrier patients should be advised breast‐feeding less than 3 months.^12^, ^13^

### 3.5 Maternal VTE prophylaxis

In general, wheelchair use in daily life is a risk factor for developing VTE, and the patients have a higher risk during pregnancy and postpartum period.^14^ Regarding this case, the patient was a pregnant woman and wheelchair user as risk factors for VTE. She was classified as a middle risk of VTE during pregnancy; therefore, non‐pharmacological prophylaxis was selected for this treatment. In the cases of pregnancy complicated with HAM/TSP, it is possible to prevent VTE during pregnancy and postpartum period by following various guidelines for the prevention of VTE during pregnancy such as RCOG Green‐top Guidelines.^15^

We presented here of one woman with HAM/TSP and her three times consecutive courses of pregnancy and childbirth without complications on her and her baby. In conclusion, the woman patients with HAM/TSP who are wheelchair users with spastic paraparesis should not be discouraged from pregnancy.

## CONFLICT OF INTEREST

None declared.

## AUTHOR CONTRIBUTIONS

HM: involved in the management of pregnancy and labor and wrote the manuscript. TK, TU, and EK: involved in management of labor. YI: involved in management of neurological symptoms during pregnancy. ES and KY: supervised the project. All authors: discussed the results and contributed to the final manuscript.

## ETHICAL APPROVAL

The study was approved by the institutional ethics committee, and published with written consent of the patient.

## Data Availability

The data that support the findings of this study are available from the corresponding author upon reasonable request.
